# Sustainable nanomedicine:green synthesis of functional nanomaterials and its applications

**DOI:** 10.3389/fchem.2026.1890004

**Published:** 2026-07-29

**Authors:** Banhi Halder, Anushka Satpathy, Kajal Kumari, Vinod Kumar Nigam

**Affiliations:** 1 Department of Bioengineering and Biotechnology, Birla Institute of Technology, Mesra, Jharkhand, India; 2 Department of Biotechnology, School of Allied Health Sciences, ARKA Jain University, Jamshedpur, Jharkhand, India

**Keywords:** antimicrobial therapy, drug delivery, functionalized nanomaterials, green synthesis, nanomedicine, regenerative medicine

## Abstract

Nanomedicine has emerged as a promising platform for targeted drug delivery, molecular diagnostics, cancer therapy, and regenerative medicine by exploiting the unique physicochemical properties of nanomaterials. However, conventional nanoparticle synthesis often relies on hazardous chemicals, energy-intensive processes, and complex purification steps that may limit biocompatibility, reproducibility, and large-scale clinical translation. Green synthesis has therefore gained increasing attention as a sustainable alternative, employing biological resources such as plant extracts, microorganisms, enzymes, and biopolymers to produce functional nanomaterials under environmentally benign conditions. These biologically derived nanoparticles exhibit enhanced surface functionality and have demonstrated applications in drug delivery, antimicrobial therapy, cancer treatment, bioimaging, biosensing, tissue engineering, and advanced biofabrication, including nano-enabled hydrogels and bioinks for 3D/4D bioprinting. Nevertheless, green synthesis should not be regarded as inherently non-toxic, as nanoparticle safety remains dependent on physicochemical characteristics, dosage, biodistribution, and biological interactions. Furthermore, challenges including batch-to-batch variability, mechanistic uncertainty, limited process standardization, long-term toxicity, and regulatory barriers continue to hinder clinical translation. This review critically examines recent advances in green synthesis, compares conventional and biological nanoparticle fabrication strategies, discusses functionalization approaches that improve therapeutic performance, and highlights current biomedical applications together with the key challenges and future perspectives for developing sustainable and clinically translatable nanomedicine.

## 1 Introduction

Nanomedicine represents a groundbreaking multidisciplinary domain bridging nanotechnology, material science, biotechnology, and medical science, where innovative nanodevices can be developed for targeted drug delivery, molecular detection, bioimaging, antibacterial therapy, and regenerative medicine. The nanomaterials are endowed with distinctive physicochemical characteristics, enhanced surface area-to-volume ratio, and quantum effects, making them more attractive candidates compared with bulk substances for precision healthcare purposes. Nanodevices have facilitated accurate disease diagnosis and treatment through improved drug solubility, cell entry, precise drug delivery, imaging, and theragnostic. Applications of nanomedicine have expanded into cancer treatments, infectious diseases, biosensors, wound healing, and tissue regeneration via 3D/4D bioprinting (Gupta et al., 2023). However, there are some challenges to the adoption of conventional nanomedicine across healthcare practices, primarily due to the cytotoxic nature of synthesized chemicals, low reproducibility rate, inconsistencies, difficulties in scaling up the production process, energy-intensive procedures, and environmentally harmful manufacturing technologies. Conventional fabrication techniques require toxic solvents, strong reduction reagents, high temperatures and pressures, and complicated purification steps, leading to high costs and safety risks to the human body and the environment (Table 1) (Gupta et al., 2023).

**TABLE 1:** Comparison of conventional and green synthesis routes for functional nanomaterials in sustainable nanomedicine.

Parameters	Conventional synthesis	Green synthesis
Typical routes	Chemical reduction, sol–gel, hydrothermal, co-precipitation, thermal decomposition, laser ablation	Plant-mediated, microbial synthesis, enzyme-assisted, biopolymer-assisted
Reducing agents	NaBH_4_, hydrazine, citrate, ethylene glycol	Polyphenols, flavonoids, proteins, enzymes, polysaccharides, microbial metabolites
Energy demand	High (heating/pressure) 80 °C–300 °C	Low (ambient conditions) 20 °C–60 °C
Biocompatibility	Moderate	Higher
Waste generation	Hazardous solvent and byproduct	Reduced waste; predominantly aqueous processes
Particle control	High monodispersity and tunability	Moderate; influenced by biological source, extract composition, and reaction conditions
Scalability	Commercially established	Pilot to emerging industrial scale
Clinical sustainability	Limited	Strong potential
Major limitation	Toxic reagents, environmental burden	Batch variability, incomplete mechanistic understanding, process standardization, and regulatory challenges

As a response to the above issues, the concept of green fabrication of functionalized nanomaterials has been gaining much popularity as a more sustainable approach compared to traditional nanofabrication. Green nanotechnology utilizes natural resources like plant extract, bacteria, fungi, algae, enzymes, polysaccharides, etc., as reducing, stabilizing, and capping agents for the formation of various nanomaterials via mild reactions. Apart from being eco-friendly and having low energy consumption during the process, such biologically-inspired approaches produce nanomaterials with high biocompatibility, biodegradability, colloidal stability, and bioactivity, which is favourable for medical application. More importantly, the presence of functional moieties in nanomaterials through biomolecule-based syntheses can significantly facilitate interactions and enhance the performance of nanomedical therapy (Ayub et al., 2025; Gupta et al., 2023; Lawal et al., 2025).

Green chemistry may minimize the use of hazardous reagents and enhance the biocompatibility of nanoparticles by utilizing biological surface capping. It would be erroneous to assume that green synthesis is inherently non-toxic. Biological toxicity associated with green-synthesized nanoparticles is highly influenced by physical and chemical properties such as size, shape, surface charge, concentration, distribution within the body, and duration of exposure. For instance, biologically synthesized silver nanoparticles can cause oxidative stress, mitochondrial dysfunction, and cytotoxicity at high concentrations (Khoramipour et al., 2026).

Significant progress has been made in green-synthesized metallic nanoparticles, metal oxides, carbon quantum dots, graphene-based materials, magnetic nanomaterials, nanozymes, nanocomposite hydrogels, and nano-bioinks, thus paving ways for more sophisticated next-generation nanomedicine. They have shown significant potentials in controlled drug delivery, antimicrobial and anticancer property, sensing and detection, fluorescence imaging, Magnetic resonance imaging (MRI) contrast enhancement, tissue regeneration, and 4D printing. Hence, the merging of green chemistry with biomedical engineering can lead to more sustainable and effective treatment in the clinic (Karnwal et al., 2024; Kumar et al., 2025).

A number of recent reviews have brought to light certain key issues in the field of nanomedicine such as theranostic gold nanoparticles, carbon-based drug carriers, magnetic nanoparticles, nanocarrier-enabled delivery of phytochemical bioactive, and nanofiber scaffold composites for regeneration medicine. In spite of these reviews being concerned with particular types of nanomaterials or single medical uses, there is still a need to provide a comprehensive review that brings together sustainable green synthesis approaches, nanoparticle functionalization, state-of-the-art diagnostics, regenerative nanomedicine, and the related translational issues.

Though many review articles have considered different aspects of nanomedicine like green nanoparticle synthesis, functionalization of nanoparticles, drug delivery, carbon-based nanomaterials, magnetic nanoparticles, and regenerative biomaterials, all these aspects have been considered separately. Recent developments in areas like multifunctional nanoplatforms, nano-biosensors, theranostic platforms, electrospun nanofibers, smart bioinks, and Artificial intelligence (AI) enabled nanomedicine have greatly increased the horizon of sustainable nanomedicine. But still there is a lack of review which integrates environmentally sustainable synthesis of nanoparticles with functionalization and biomedical applications along with the challenges related to its clinical translation. This review is not similar to other reviews that have covered either a particular group of nanomaterials or only one biomedical application. The current review has brought together the latest advancements in sustainable synthesis, functionalization of nanoparticles, diagnostic and therapeutic applications, regenerative medicine, and bioprinting in order to create a unified perspective on sustainable nanomedicine. Also, this review highlights the challenges facing the field in terms of reproducibility, nanotoxicology, standardization of the processes, scaling-up, and regulatory approval, and emerging trends in sustainable nanomedicine including multifunctional theranostic, nanocarrier-delivery of bioactive compounds, electrospun nanofibers, and smart nanomaterials for precision medicine.

Consequently, this review highlights the recent developments in sustainable nanoparticle synthesis, compares the traditional and green methods of nanoparticle synthesis, describes ways to improve the effectiveness of nanoparticles through functionalization, outlines their biomedical applications, and emphasizes the important issues that should be resolved in order to promote green nanomedicine.

## 2 Conventional nanomedicine

### 2.1 Synthesis of nanomaterials

The evolution of traditional nanomedicine has largely been fuelled by physical chemistry-based strategies. It allows the control of particle dimensions, morphology, and surface properties. These methods form the fundamental principles of nanocarrier development ranging from metallic nanoparticles to metal oxides, liposomes, dendrimers, and polymers used in therapeutics and diagnostics (Patra et al., 2018).

An important aspect of conventional nanomedicine involves understanding the different conventional synthesis processes of nanomaterials. A comparative summary of major conventional synthesis techniques is highlighted in Table 2. Among all the methods used, chemical reduction is one of the most popular techniques. It is one of the key processes involved in the production of noble metal nanoparticles like gold and silver (Daniel and Astruc, 2004). During this procedure, metal ions are converted into nanoparticles using chemical reducers, for example, sodium borohydride, hydrazine and citrate derivatives. Although this method facilitates fast nucleation, the major disadvantage that comes with the use of chemicals is their residue and the potential toxicity (Gupta et al., 2023; Iravani, 2011). Some of the other methods used to synthesize nanoparticles include the sol-gel method, and the hydrothermal or solvothermal method. The sol-gel method involves hydrolytic and polycondensation reactions to produce oxide nanomaterials including TiO_2_ and SiO_2_, whereas the hydrothermal method utilizes high temperatures and pressures to prepare nanomaterials with high crystallinity (Kharisov et al., 2014). Co-precipitation is another technique used for preparing magnetic nanoparticles such as Fe_3_O_4_ because it is relatively simple and scalable, but it lacks precise size control (Kharisov et al., 2014). Other physical methods like laser ablation and thermal decomposition provide more options for the synthesis process, which are beneficial in terms of purity and monodispersity. Nonetheless, these approaches are relatively energy-consuming and involve complicated equipment, making them less accessible (Iravani, 2011).

**TABLE 2:** Conventional nanoparticle synthesis methods in nanomedicine.

Method	Principle	Common materials	Advantages	Limitations
Chemical reduction	Reduction of metal salts using strong reducing agents	Au, Ag NPs	Fast, controllable size	Toxic reagents, residues
Sol–gel	Hydrolysis and condensation of precursors	TiO_2_, SiO_2_	High purity, uniformity	Multi-step, solvent use
Hydrothermal/Solvothermal	High temp and pressure crystallization	ZnO, Fe_3_O_4_	Crystalline, controlled morphology	Energy-intensive
Co-precipitation	Simultaneous precipitation of ions	Fe_3_O_4_, metal oxides	Simple, scalable	Poor size control
Thermal decomposition	Heat-induced precursor breakdown	Metal NPs	Monodispersity	High temperature, toxic solvents
Laser ablation	Physical vaporization using laser	Au, Ag	No chemical contamination	Expensive, low yield

In biomedical research, sensitive nanoparticles like liposomes, polymeric nanoparticles, and micelles can be prepared by methods such as emulsification, nanoprecipitation, and solvent evaporation (Patra et al., 2018). They are extremely useful because they have the potential to encapsulate drugs and increase their bioavailability. Surface modification approaches like PEGylation and ligand functionalization are commonly used to increase circulation times and targeting capabilities (Kumar et al., 2025). Even though these conventional methods have advantages of flexibility and controllability, the limitation still exists. Traditional approaches of synthesis are characterized by multiple steps, stringent reaction requirements, and rigorous purification, all of which require a lot of resources and are time intensive (Gupta et al., 2023).

### 2.2 Limitation of conventional nanomedicine synthesis

Although traditional nanotechnology has made remarkable contributions to targeted treatment and diagnostics, there still exist various drawbacks associated with its widespread medical application. For example, toxicity becomes a major issue due to the utilization of toxic chemical reagents in the production process and surface contamination in the form of impurities. These toxic effects further induce oxidative stress, inflammation, and adverse biological responses in biological systems (Gupta et al., 2023). In close association with this is the question of sustainability of the environment. Traditional methods of synthesis use toxic solvents, consume high amounts of energy, and produce dangerous waste products. This makes not only the environmental impact greater, but also makes production costs higher, thus making mass-production unsustainable (Iravani, 2011).

**FIGURE 1:**
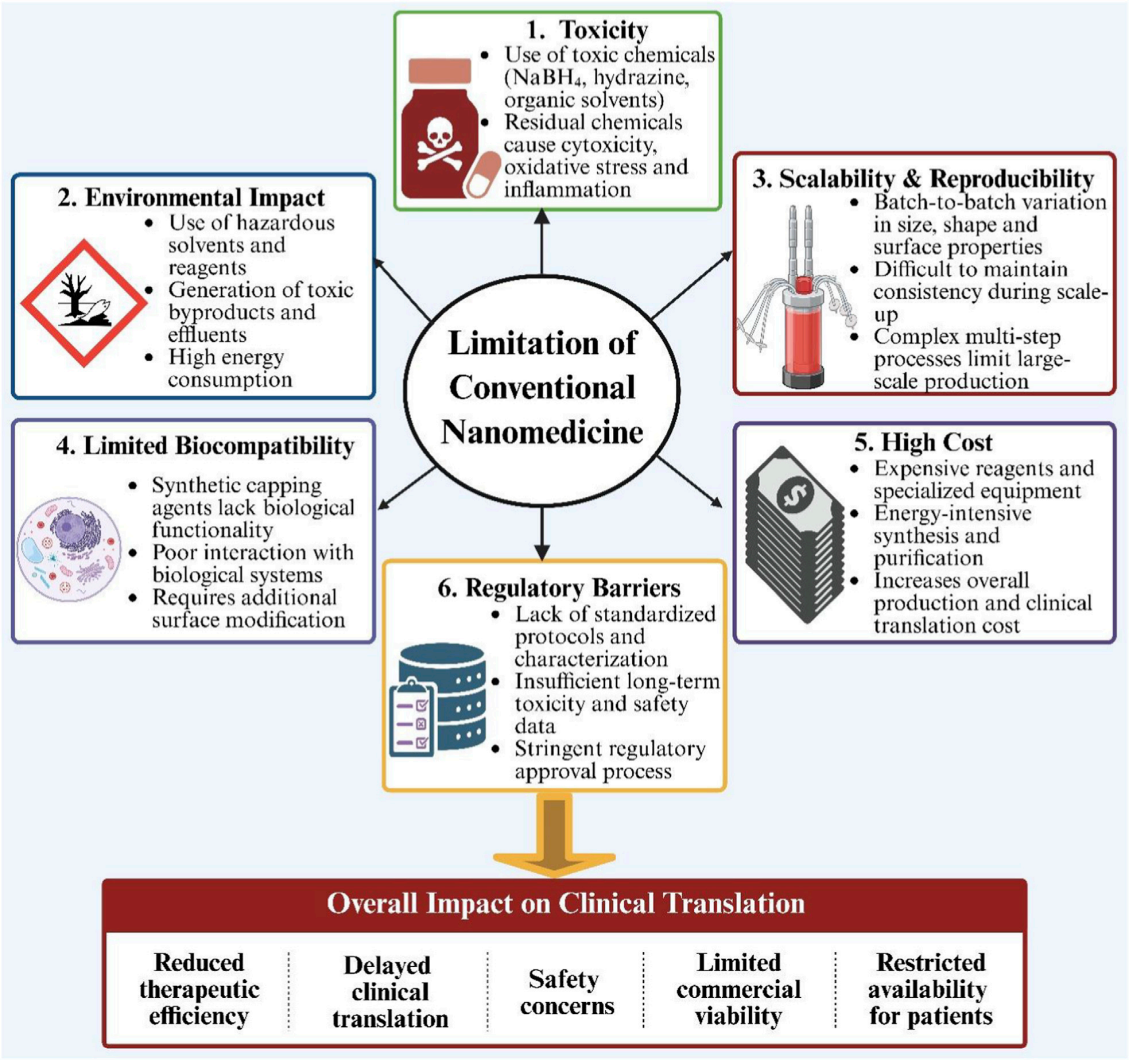
Key limitations of conventional nanomedicine.

Another critical constraint lies in the scalability and reproducibility of the conventional synthesised nanomedicines. While laboratory synthesis offers the possibility of controlled fabrication, the difficulty comes when trying to reproduce the same results at a larger scale. The differences in batch-to-batch production could affect the efficacy of the therapy being developed and its regulatory outcomes. From the functional point, traditional nanomaterials usually lack sufficient biocompatibility and thus necessitate surface modifications for enhancing their biological interactions (Mitragotri et al., 2015). This modification further adds the complexity and variability of nanomedicines. Also, issues such as the absence of standardization, inadequate safety information, and strict regulations have always prevented nanomedicines from being utilized in common healthcare practices (Bobo et al., 2016; Mitragotri et al., 2015). These interconnected challenges are shown in Figure 1.

## 3 Green synthesis of nanomaterials

Increasing concerns regarding the environmental impact and sustainability of conventional nanofabrication have accelerated the development of green synthesis approaches. By integrating green chemistry principles with nanotechnology, green synthesis has emerged as a key strategy for advancing sustainable nanomedicine. Green nanomaterial synthesis combines the concepts of green chemistry and nanotechnology. Green synthesis uses biological resources such as plant extracts, microorganisms, enzymes, and biopolymers to produce nanoparticles under environmentally benign conditions (Ayub et al., 2025; Iravani, 2011).

Plant-mediated synthesis is one of the highly explored methods of green synthesis. In this method, plant secondary metabolites like polyphenols, flavonoids, alkaloids, and terpenoids act as reducing agents for the formation and stabilization of nanoparticles (Ayub et al., 2025). This twofold process renders the use of additional chemicals redundant and gives rise to highly bioactive nanoparticles. Likewise, can be utilized as biological systems to produce nanoparticles by virtue of enzyme-mediated reduction processes (Lawal et al., 2025; Saxena et al., 2025). In addition to bio-synthesis system, enzymatic and biopolymer/biomolecule assisted nanoparticle synthesis methods provide better control over nanoparticle formation. Biomolecules such as proteins, chitosan, cellulose, and dextran serve as natural capping agents on nanoparticles, giving rise to functional groups with improved colloidal stability and bio-compatibility (Karnwal et al., 2024). Compared with other biological approaches, plant-mediated and microbial synthesis are currently the most widely investigated strategies for biomedical applications (Ayub et al., 2025; Lawal et al., 2025). Plant-mediated synthesis is simple, cost-effective, and can be readily scaled up because plant extracts contain phytochemicals that function as both reducing and stabilizing agents (Ayub et al., 2025). However, variations in plant species, growing conditions, and extract composition may influence nanoparticle size and reproducibility (Ayub et al., 2025). In comparison, microbial synthesis generally provides better control over particle morphology through enzyme-mediated reduction mechanisms (Lawal et al., 2025; Saxena et al., 2025). However, it requires sterile culture conditions and additional purification steps, making large-scale production more challenging (Lawal et al., 2025). Overall, plant-mediated synthesis appears more suitable for sustainable large-scale production, whereas microbial synthesis is advantageous when better control over nanoparticle characteristics is required (Ayub et al., 2025; Lawal et al., 2025). A comparison of the major green synthesis strategies and their translational challenges is summarized in Table 3.

**TABLE 3:** Comparison of plant-mediated and microbial green synthesis strategies for biomedical nanomaterials.

Parameter	Plant-mediated synthesis	Microbial synthesis
Biological source	Plant extract	Bacteria/Fungi/Algae
Cost	Low	Moderate
Ease of synthesis	Simple	Requires sterile culture
Particle uniformity	Moderate	Good
Scale-up	Easier	More difficult
Major bottleneck	Variability of phytochemicals	Purification and biosafety
Clinical maturity	High	Moderate

This comparison highlights that plant-mediated and microbial synthesis each present distinct advantages and translational challenges. Therefore, the selection of an appropriate synthesis strategy should be guided by the intended biomedical application and the desired physicochemical properties of the nanoparticles.

Notably advantages of green nanoparticle synthesis is the fact that these materials are compatible with biological systems and exhibit lower toxicity. As by the name “green-synthesis” there are no hazardous chemicals used during production which minimizes the adverse toxicity effect. The green nanoparticles are also more effective because of enhanced bioactivity. Their surfaces possess functional groups that enable higher efficiency in cellular delivery/uptake and target recognition with enhanced therapeutic performance (Karnwal et al., 2024). These nanoparticles also show improved antimicrobial, anticancer, and catalytic activities because of their unique surface chemistry (Lawal et al., 2025). The benefits of green synthesis can be fully understood only when compared with conventional synthesis approaches. As summarized in Table 4, green-synthesized nanoparticles generally exhibit improved biocompatibility and environmental sustainability while maintaining comparable antimicrobial performance.

**TABLE 4:** Comparison of conventionally synthesized and green-synthesized nanoparticles for antimicrobial applications.

Feature	Conventional synthesized nanoparticles	Green-synthesized nanoparticles
Synthesis route	Chemical reduction, sol–gel, hydrothermal methods	Plant-, microbial-, or enzyme-mediated synthesis
Reducing/stabilizing agents	Sodium borohydride, hydrazine, citrate, synthetic surfactants	Phytochemicals, microbial metabolites, enzymes, polysaccharides
Surface chemistry	Synthetic capping agents	Natural biomolecule capping
Antimicrobial activity	Effective against a broad range of pathogens	Comparable or enhanced activity due to bioactive surface molecules
Biocompatibility	Moderate; may require additional surface modification	Generally higher because of biomolecule-mediated surface functionalization
Residual toxicity	Higher risk due to chemical residues	Lower because hazardous reducing agents are avoided
Environmental impact	High energy demand and hazardous waste generation	Low energy requirement and environmentally friendly process
Translational challenge	Chemical toxicity and environmental concerns	Batch-to-batch variability and process standardization

**FIGURE 2:**
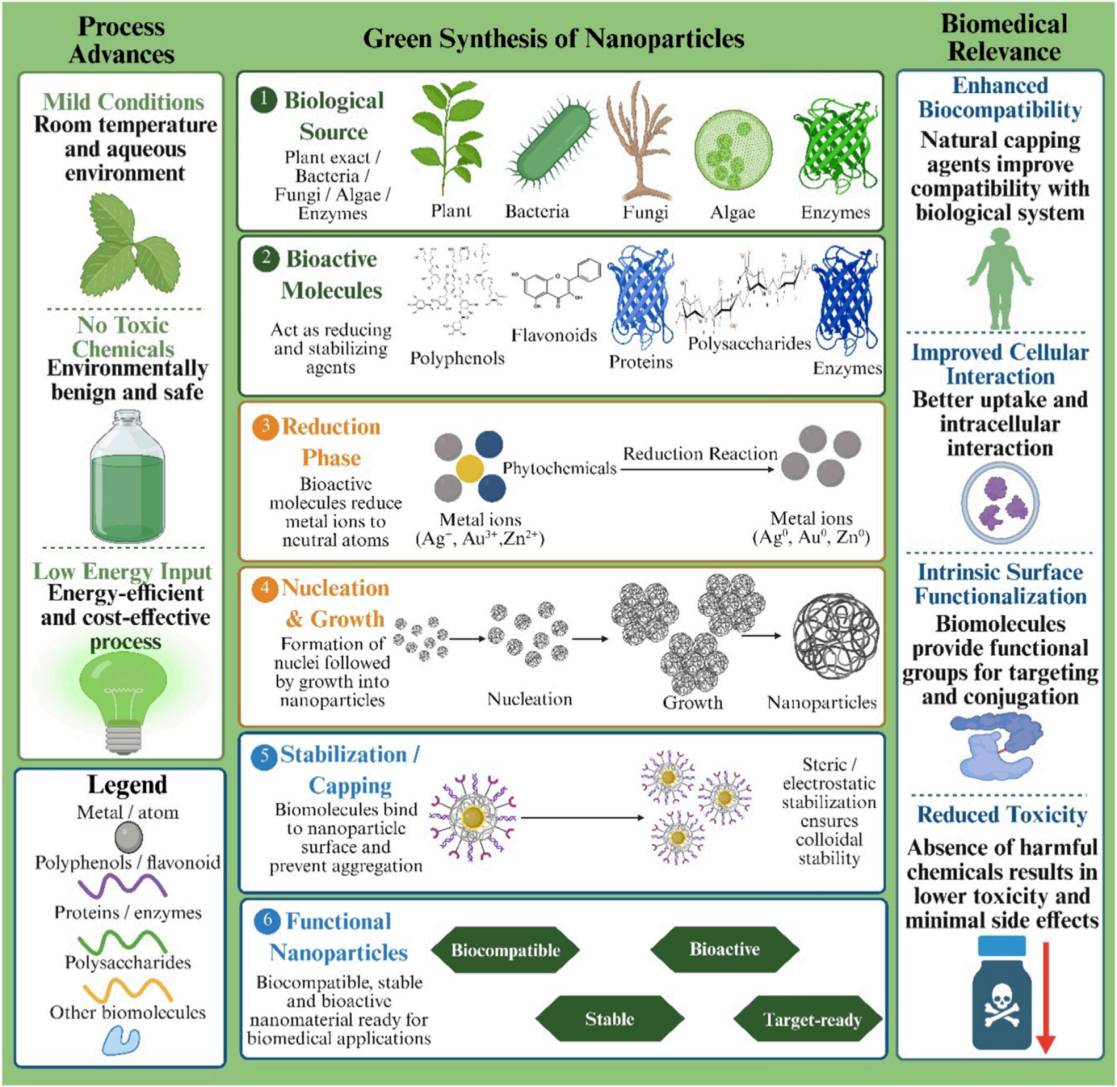
Mechanism and biomedical relevance of green nanoparticle synthesis.

Green synthesis methods have significantly low energy consumption and low levels of waste generation because they are conducted at room temperature with water solutions (Ayub et al., 2025). These characteristics not only improve the biomedical performance of nanomaterials but also reinforce the concept of sustainable nanomedicine by reducing chemical waste, lowering energy consumption, and promoting environmentally responsible manufacturing. Green synthesis also has its drawbacks despite the aforementioned benefits. Variability in biological sources, differing extract compositions, and unclear reaction mechanisms lead to inconsistent results. Additional challenges include controlling particle size distribution, scaling up for industrial applications, and obtaining regulatory acceptance (Gupta et al., 2023; Lawal et al., 2025). Therefore, overcoming these challenges will be essential for translating green synthesis from laboratory research into sustainable nanomedicine with real clinical and industrial impact. An overview of mechanism of green nanoparticle synthesis and its biomedical relevance is shown in Figure 2.

Advances in green nanomedicine in recent years: Green nanomedicine has moved from traditional synthesis of nanoparticles using plants to development of multifunctional and intelligent nanoparticles, in recent years. The most important among these advances are enzyme-mediated synthesis, AI-based optimization, theranostic nanoparticles, wearables biosensors, and smart bioinks for regenerative medicine. The key advances and their medical importance are detailed in Table 5.

**TABLE 5:** Recent advances in sustainable nanomedicine and green-synthesized functional nanomaterials.

Recent advance	Representative findings	Biomedical significance	Key references
Enzyme-assisted green synthesis	Improved control over particle size and colloidal stability compared with conventional plant extracts	Enhanced reproducibility and biocompatibility	Lawal et al. (2025), Saxena et al. (2025)
Al-assisted optimization of nanoparticle synthesis	Machine learning used to optimize reaction parameters and nanoparticle characteristics	Better process optimization and reduced experimental time	Kumari et al. (2024), Mallick et al. (2025)
Continuous-flow/microfluidic synthesis	Continuous production improves scalability and batch consistency	Facilitates industrial translation	Kumari et al., 2024 (2025)
Multifunctional theranostic nanoparticles	Simultaneous drug delivery, imaging and target therapy	Precision medicine and personalized treatment	Khoramipour et al. (2026)
Wearable nanobiosensors	Integration with AI and loT for real-time monitoring	Point-of-care diagnostics	Mallick et al. (2025), Thiruvengadam et al. (2025)
Smart nano-enabled bioinks	Stimuli-responsive bioinks for 3D/4D bioprinting	Advanced tissue engineering and regenerative medicine	Municoy et al. (2025)

Although considerable progress has been made, translating green nanomaterials into clinical practice still requires better reproducibility, clearer insight into synthesis mechanisms, and scalable manufacturing technologies. Continued efforts in these areas will strengthen the development of sustainable nanomedicine and support its wider biomedical application.

## 4 Fundamentals of nanoparticle functionalization

Nanoparticles have physicochemical properties that distinguish them from ordinary substances owing to their nanometre size, high surface-to-volume ratio, and quantum confinement effects (Asha and Narain, 2020). The physicochemical properties are responsible for making metal, semiconductors, and magnetic nanoparticles display outstanding optical, magnetic, catalytic, and electronic properties. Nevertheless, nanoparticles are intrinsically thermodynamically unstable and exhibit the tendency to aggregate because of surface free energy and van der Waals forces (Aminzai et al., 2024). This limits the ability of the nanoparticles to disperse and function effectively. Therefore, functionalizing the surfaces of nanoparticles has become necessary in order to increase stability, hydrophobicity, biocompatibility, and surface reactivity (Sanità et al., 2020).

### 4.1 Physicochemical basis

Nanoparticle functionalization is based on the physicochemical principles of the increased reactivity of the surface atoms due to their interaction at the interface between nanoparticles and other materials (Wieszczycka et al., 2021). These atoms have uncoordinated bonding sites and dangling bonds for adsorption, coordination, chemical bonding, and interactions with the molecules in the environment (Cai et al., 2017). Functionalization of nanoparticles can be explained by the reduction of surface free energy and prevention of nanoparticle agglomeration using the principles of electrostatic, steric, or electrostatic stabilization (Navarro-Tovar et al., 2023). In particular, charged functional groups provide repulsion, while polymer molecules serve as a barrier that results in improved colloidal stability. Other characteristics of nanoparticles that change during functionalization include hydrophilicity/hydrophobicity, aqueous dispersibility, charge transfer, adsorption properties, and catalytic activity (Sampaio and Dalmaschio, 2024).

### 4.2 Mechanisms of surface stabilization

The effectiveness of nanoparticle functionalization originates from the fundamental chemistry occurring at nanoparticle interfaces. Surface atoms in nanoparticles possess unsaturated coordination environments and dangling bonds, making them highly reactive. These reactive surface atoms interact with ligands, polymers, and biomolecules through covalent bonding, electrostatic attraction, hydrogen bonding, hydrophobic interactions, π–π stacking, or coordination chemistry (Gorohovs and Dekhtyar, 2025). The stabilization of functionalized nanoparticles generally occurs through three major mechanisms: electrostatic stabilization, steric stabilization, and electrosteric stabilization. In electrostatic stabilization, charged functional groups such as carboxyl, amine, sulfate, and phosphate groups are introduced onto the nanoparticle surface, generating repulsive electrostatic interactions between adjacent particles. These repulsive forces prevent nanoparticle aggregation and maintain colloidal dispersion stability in aqueous systems (Mudunkotuwa and Grassian, 2015). In steric stabilization, polymeric ligands such as polyethylene glycol (PEG), polyvinylpyrrolidone (PVP), and other macromolecules form a protective barrier around the nanoparticle surface (Tadros, 2013). The polymer chains generate steric barriers that prevent close particle-particle interactions and suppress agglomeration. Thereby enhancing dispersion stability even under physiological conditions (Bijlard et al., 2017; Fang et al., 2009; Grubbs, 2007). Despite its widespread use, PEGylation is associated with certain limitations, commonly referred to as the “PEG dilemma.” Although polyethylene glycol (PEG) significantly enhances nanoparticle stability, prolongs systemic circulation, and reduces nonspecific protein adsorption by forming a hydrophilic stealth layer, it may simultaneously decrease cellular internalization and intracellular drug delivery due to reduced interactions with target cell membranes. Furthermore, repeated administration of PEGylated nanoparticles has been reported to induce the production of anti-PEG antibodies, leading to the accelerated blood clearance (ABC) phenomenon, complement activation, and hypersensitivity reactions, thereby compromising therapeutic efficacy and safety. These limitations have prompted the development of alternative stealth strategies, including zwitterionic polymers, polysarcosine, poly (2-oxazoline)s, and biomimetic surface coatings, which aim to retain prolonged circulation while improving cellular uptake and reducing immunogenicity (Garay et al., 2012; Knop et al., 2010; Kozma et al., 2020). Electrosteric stabilization combines both electrostatic repulsion and steric hindrance, providing superior long-term stability in complex biological and environmental systems. In this mechanism, charged polymeric ligands simultaneously generate surface charge repulsion and steric barriers, resulting in enhanced resistance against aggregation under varying pH and ionic strength conditions. Additionally, surface engineering through these stabilization strategies significantly influences adsorption behaviour, catalytic activity, light adsorption, charge separation efficiency, and interactions with biomolecules, thereby forming the fundamental basis for rational nanoparticle design and biomedical functionality (Fritz et al., 2002; Markiewicz et al., 2018).

### 4.3 Surface chemistry and instability

#### 4.3.1 Surface energy and instability

Nanoparticles possess high surface energy because a large proportion of their atoms are located at the surface rather than within the interior bulk structure. Surface atoms possess unsaturated coordination sites and dangling bonds, resulting in enhanced surface reactivity. Major consequences of high surface energy include agglomeration, Ostwald ripening, surface oxidation, ligand desorption, and catalytic inefficiency (Bhattacharjee and Prasad, 2023; Holec et al., 2020).

#### 4.3.2 Surface charge and colloidal stability

The colloidal stability of nanoparticles is strongly governed by zeta potential, surface charge density, interfacial interactions, and the physicochemical properties of the surrounding medium. Surface functionalization introduces ionic or polymeric ligands that reduce nanoparticle aggregation and improve dispersion stability under physiological conditions (Bent, 2002). However, nanoparticle stability is highly sensitive to environmental parameters such as pH, ionic strength, electrolyte concentration, and biomolecular adsorption.

Under physiological conditions, compression of the electrical double layer may weaken electrostatic repulsion and promote particle aggregation, thereby limiting the effectiveness of purely electrostatic stabilization mechanisms. In contrast, steric stabilization using polymeric ligands such as PEG provides enhanced resistance against nonspecific protein adsorption and improves systemic circulation stability. Electrosteric stabilization, which combines steric hindrance with electrostatic repulsion, offers superior colloidal stability in biologically complex environments and is therefore highly advantageous for nanomedicine applications requiring prolonged physiological stability and controlled biointeractions.

### 4.4 Classification of antimicrobial nanoparticles and their functionalisation

Different classes of nanoparticles exhibit distinct therapeutic mechanisms depending on their composition, surface chemistry, and functionalization strategies. Surface modification of these nanoparticles significantly improves their colloidal stability, biocompatibility, targeting efficiency, drug loading capacity, and therapeutic performance. Functionalized nanoparticles have therefore gained considerable attention in antimicrobial therapy, cancer treatment, bioimaging, targeted drug delivery, and theranostic applications. The major classes of nanoparticles, their antimicrobial and anticancer mechanisms, and biomedical significance are summarized in Table 6.

**TABLE 6:** Classification of different nanoparticles and their major antimicrobial and anticancer applications along with their underlying therapeutic mechanisms and biomedical significance.

Nanoparticle class	Representative nanoparticles	Mechanism of antimicrobial action	Mechanism of anticancer action	Key biomedical significance	References
Noble Metal Nanoparticles	AuNPs, AgNPs	Cell membrane disruption, ROS generation, protein denaturation	Photothermal destruction, apoptosis induction, targeted delivery	Broad-spectrum antimicrobial and theranostic applications	Balestri et al. (2023)
Metal Oxide Nanoparticles	ZnO, TiO_2_, CuO	Oxidative stress generation and microbial enzyme inhibition	ROS-mediated cancer cell toxicity and photocatalytic therapy	Useful in antimicrobial coatings and cancer therapeutics	Generalova and Dushina (2025)
Magnetic Nanoparticles	Fe_3_O_4_, CoFe_2_O_4_	Magnetic pathogen separation and antimicrobial carrier systems	Magnetic hyperthermia and MRI-guided drug targeting	Simultaneous imaging and therapy applications	Shabatina et al. (2023)
Quantum Dots	CdSe, CdTe quantum dots	Photoinduced microbial inactivation	Fluorescent tumor imaging and photodynamic therapy	High sensitivity bioimaging and diagnostics	Hanmante et al. (2025), Shen et al. (2025)
Carbon-Based Nanomaterials	CNTs, Graphene oxide	Physical membrane piercing and oxidative damage	Drug transport and photothermal tumor ablation	High surface area for multifunctional therapeutic systems	Hussen et al. (2024)
Polymeric Nanoparticles	PLGA, Chitosan nanoparticles	Sustained antimicrobial drug release	Controlled chemotherapeutic release and tumor targeting	Enhanced biocompatibility and reduced drug toxicity	Harini and Perumal (2025), Olmos and González-Benito (2021)

ROS, reactive oxygen species; PLGA, Poly (lactic-co-glycolic acid); AuNPs, Gold nanoparticles; AgNPs, Silver nanoparticles; TiO_2_, titanium dioxide; CdSe, Cadmium selenide; CdTe, Cadmium telluride; CNTs, Carbon nanotubes.

#### 4.4.1 Metallic nanoparticles

Metallic nanoparticles are most researched types of nanomaterials because of their special optical, electronic, catalytic, and physical-chemical characteristics that originate from quantum confinement effect and high surface area to volume ratio. This includes gold nanoparticles (AuNPs), silver nanoparticles (AgNPs), and platinum nanoparticles, which have a wide range of applications in biomedicine, detection, catalysis, and environmental protection (Serna-Gallén and Mužina, 2026). AuNPs have very good biocompatibility and strong surface plasmon resonance (SPR), and therefore, they can be applied in biosensors, bioimaging, and drug delivery. AgNPs have high antimicrobial activity and electrical conductivity, which makes them suitable for coating applications and biomedical purposes such as medical implants and water purification. Surface plasmon resonance is one of the features of metallic nanoparticles that leads to the development of tunable optical adsorption and scattering. Surface plasmon resonance can be varied according to particle size and shape. The reactive surface area of contributes to enhanced catalytic activity and electrical conductivity. This tends to aggregate and oxidize, thus requiring surface modification through functionalization with various ligands, polymers, or biomolecules. This makes metallic nanoparticles an essential material for nanotechnology (Aguilar-Garay et al., 2024).

#### 4.4.2 Semiconductor nanoparticles

Semiconductor nanoparticles are an important class of nanomaterials exhibiting unique optical, electronic, and photochemical properties due to quantum confinement effects and size-dependent electronic structures. Common examples include cadmium selenide (CdSe) and cadmium telluride (CdTe) quantum dots, zinc oxide (ZnO) nanoparticles, and titanium dioxide (TiO_2_) nanoparticles, which are widely used in biomedical, photocatalytic, environmental, and optoelectronic applications (Koole et al., 2014).

CdSe and CdTe quantum dots are well known for their strong fluorescence and tunable bandgap energies, were particle size controls emission wavelength and optical behaviour. This makes them very important for applications such as bioimaging, biosensing, fluorescence labelling, and photovoltaics. The two materials, ZnO and TiO_2_, have shown great potential for photocatalysis and charge separation. Under illumination, they form electron-hole pairs that react in oxidation-reduction reactions to decompose pollutants, kill bacteria, and convert solar energy. TiO_2_ particles are favoured because of their chemical stability and high oxidative power while ZnO nanoparticles have the ability to absorb UV radiation and inhibit bacterial growth (Surana et al., 2014). In addition to that, semiconductor nanoparticles show effective electron transfer and high surface activity because of their high surface area to volume ratio. On the other hand, they have a tendency to agglomerate and develop surface defects, which is why they need to be stabilized or functionalized with polymers, ligands, or surfactants (Khairutdinov, 1998).

#### 4.4.3 Magnetic nanoparticles

Of the many forms of nanomaterials, there are some specific forms that exhibit magnetic properties due to their small size and magnetic domains. Of these magnetic nanoparticles, there are two types which are usually employed, namely, iron oxide (Fe_3_O_4_) and cobalt ferrite (CoFe_2_O_4_). Magnetic nanoparticles exhibit magnetic activity and large surface areas. They are controllable in an external magnetic field. One of the major characteristics of magnetic nanoparticles is super para-magnetism, whereby nanoparticles show a high level of magnetism when exposed to an external magnetic field; however, they do not retain any residual magnetism when the magnetic field is withdrawn. This helps to prevent aggregation, thus ensuring high stability of the colloid, which makes them ideal for biomedical applications. Fe_3_O_4_ nanoparticles have been widely used in MRI for their role in increasing the sensitivity of images. Furthermore, magnetic nanoparticles are widely used in targeted drug delivery, hyperthermia, biosensing, and bioseparation methods (Gulati and Yadav, 2025).

With the recent developments in this field, there has been an increase in the application of magnetic nanoparticles beyond MRI contrast agents into multifunctional theranostic devices that facilitate targeted drug delivery, magnetic hyperthermia, biosensing, and image-guided therapy. It is possible to use surface-functionalized magnetic nanoparticles that respond to an external magnetic field for specific targeting without exposing the whole body to drugs (Najafi et al., 2026; Riaz et al., 2025).

Magnetic separation is another significant use of magnetic nanoparticles since external magnetic fields can help in fast and selective separation of specific molecules, cells, proteins, and pollutants from complicated mixtures. In addition, surface modification increases their compatibility, stability, and specificity, thus widening the range of their applications in nanomedicine, catalysis, water purification, and environmental remediation (Ahmad et al., 2022).

## 5 Types of nanoparticle functionalization

### 5.1 Covalent functionalization

Covalent functionalization is commonly used methods for modifying the surface of nanoparticles, which entails the creation of covalent bonding between functional molecules and the surface of nanoparticles. This method plays an especially vital role in making nanoparticles more stable, biocompatible, dispersible, and target specific (Kaymaz et al., 2023). The process is normally initiated through surface activation, during which the reactive functional groups such as hydroxyl, carboxyl, amine or thiol groups are formed on the surface of the nanoparticle. Following that, nucleophilic or electrophilic reactions take place between the surface and the functional ligands, leading to the creation of covalent bonds and stabilization of the surface (Singh et al., 2024).

One of the biggest benefits of covalent functionalization is that it allows for very stable and irreversible binding of ligands, thus providing for a decreased risk of ligand desorption and increased stability of colloids in very tough environmental conditions. Furthermore, such modification allows for greater precision of surface chemistry and provides for the ability to conjugate drugs, biomolecules, polymers, or other targeting molecules. On the other hand, covalent functionalization typically requires very complicated procedures and might change optical, electronic, or catalytic properties of nanoparticles. Covalent bonding in some conductive nanomaterials might interrupt electron transport and reduce conductivity. Nevertheless, covalent functionalization is most efficient approaches to modifying nanoparticles (Heuer-Jungemann et al., 2019).

#### 5.1.1 Thiol-based functionalization

Thiols-based functionalization is most efficient ways of covalent functionalization to modify noble metal nanoparticles, especially gold and silver nanoparticles, because of the high attraction of sulphur-containing thiols towards metal surfaces. In this process, sulfur atoms donate their lone pair electrons to metal atoms, forming strong bonds between Au-S or Ag-S. Such interaction results in very stable surface modification and formation of self-assembled monolayer on the surface of nanoparticles. Thiol functionalization makes nanoparticles more stable, dispersed, and biocompatible, and allows further conjugation with biomolecules, drugs, or targeting molecules. Because of such properties, thiols-functionalized nanoparticles find wide applications in biosensors, drug delivery systems, bio-imaging, and self-assembled nanostructures (Botcha and Narayana, 2025).

#### 5.1.2 Silanization

Silanization is a common method of covalent functionalization for modifying surfaces of oxide nanoparticles like silica, TiO_2_, and Fe_3_O_4_ nanoparticles. Silane coupling reagents like (3-Aminopropyl) triethoxysilane (APTES), Tetraethyl orthosilicate (TEOS), and 3-Mercaptopropyltrimethoxysilane (MPTS) hydrolyze to give silanol groups, which then condense with hydroxyl groups that are found on the surfaces of nanoparticles, giving rise to stable Si-O-M bonds. Silanization increases hydrophilicity, colloidal stability, surface reactivity, and bioconjugation. The process also introduces reactive groups for binding biomolecules, polymers, and drugs onto the nanoparticle surface (Huang, 2025).

#### 5.1.3 Amidation and esterification

Amidation and esterification represent some of the most crucial covalent functionalization approaches through which carboxyl-functionalized nanoparticles interact with amine or alcohol groups. Usually, activation using 1-Ethyl-3-(3-dimethylaminopropyl) carbodiimide (EDC)/N-Hydroxy succinimide (NHS) forms reactive species that can easily form stable amides and esters with biological molecules. This approach is widely applied in the coupling of proteins, immobilization of antibodies, enzymes, and drug delivery (Guo et al., 2020).

#### 5.1.4 Click chemistry

Click chemistry is a very selective and efficient way of covalent functionalization which is extensively used in nanoparticle surface modification. In many click reactions, azide-alkyne cycloaddition is the most frequently used because of its selectivity and biorthogonality. The main advantage of this type of click chemistry is that it allows one to quickly form a covalent bond in mild conditions with almost no side reactions. Click chemistry gives an excellent opportunity for controlling the surface modifications of particles, and it is perfectly adapted for biological molecules (Bhatia et al., 2025).

### 5.2 Non-covalent functionalization

Non-covalent functionalization is an important nanoparticle surface modification strategy that relies on weak intermolecular interactions rather than permanent covalent bond formation. This approach involves interactions such as electrostatic attraction, hydrogen bonding, π–π stacking, hydrophobic interactions, and van der Waals forces to adsorb functional molecules onto nanoparticle surfaces. Electrostatic interactions occur between oppositely charged species, whereas hydrogen bonding involves interactions between hydrogen donor and acceptor groups. π-π stacking is particularly significant in carbon-based nanomaterials such as graphene and carbon nanotubes, where aromatic molecules interact with delocalized π-electron systems. Hydrophobic interactions facilitate self-assembly of hydrophobic ligands in aqueous media, while weak van der Waals forces contribute to stabilization of adsorbed molecules (Rabiee and Rabiee, 2025).

A major advantage of non-covalent functionalization is the preservation of the intrinsic structural, optical, and electronic properties of nanoparticles since the core surface remains chemically unaltered. Additionally, this method generally involves simpler synthesis procedures, mild reaction conditions, and reversible interactions, making it suitable for dynamic and stimuli-responsive systems. However, non-covalent interactions are comparatively weaker than covalent bonds, resulting in lower long-term stability and possible ligand displacement under changing environmental conditions such as pH, temperature, or ionic strength. Despite these limitations, non-covalent functionalization remains highly valuable in biosensing, drug delivery, and nanobiotechnology applications (Ahmad et al., 2022).

### 5.3 Ligand exchange functionalisation

Ligand exchange is widely used nanoparticle functionalization strategies in which existing surface ligands are replaced by new ligands possessing stronger binding affinity toward the nanoparticle surface. This approach is particularly important for modifying nanoparticle solubility, stability, biocompatibility, and surface functionality. For example, hydrophobic oleic acid-coated nanoparticles synthesized in organic solvents can be transformed into hydrophilic nanoparticles through exchange with PEG, dopamine, or silane-based ligands, enabling their dispersion in aqueous and physiological media (Vo, 2024).

Ligand exchange typically starts with the diffusion of new ligands to the surface of the nanoparticle and is followed by competitive adsorption with existing ligands. Then surface displacement takes place whereby weak ligands are displaced by strong coordinating ligands, leading to surface modification and stabilization of the new nanoparticles. Ligand exchange efficiency is highly dependent on a number of factors such as ligand affinity, pH, solvent polarity, steric hindrance, and temperature among others. Efficient ligand exchange can be achieved by having optimal ligand-surface affinity and reaction conditions. Ligand exchange represents a flexible approach towards modifying nanoparticle surfaces for various biomedical, catalytic, sensing, and environmental applications (Le Goas et al., 2022).

### 5.4 Polymer functionalization

The process of polymer functionalization entails the modification of the surface of nanoparticles using natural or artificial polymers to enhance their stability, dispersion, hydrophilicity, and biocompatibility without allowing them to aggregate. The modification ensures that the nanoparticles are resistant to environmental and physiological conditions and allows for the addition of more compounds (Komsthöft et al., 2022). Various types of polymer functionalization are employed depending on the application.

#### 5.4.1 PEGylation

PEGylation involves the attachment of PEG chains onto nanoparticle surfaces to improve hydrophilicity, colloidal stability, and biocompatibility. PEG coatings reduce protein adsorption and immune recognition, thereby enhancing blood circulation time and stability in physiological environments (Suk et al., 2016).

#### 5.4.2 Polymer grafting

Polymer grafting involves the chemical bonding of polymers onto the nanoparticle surface using the “grafting to” or “grafting from” methods. The process improves the stability, functionalization of the surface, and reactivity to environmental conditions like temperature or pH.

#### 5.4.3 Polyelectrolyte coating

Polyelectrolyte coating involves adsorption of charged polymers onto nanoparticle surfaces through electrostatic interactions. These coatings improve colloidal stability, surface charge control, and biomolecule immobilization capabilities for biomedical and sensing applications.

#### 5.4.4 Hydrogel functionalization

Hydrogel functionalization incorporates nanoparticles within hydrogel matrices or hydrogel coatings to improve water retention, controlled release behaviour, and biocompatibility. These systems are widely used in tissue engineering and drug delivery applications.

#### 5.4.5 Biopolymer coatings

Biopolymer coatings using natural polymers such as chitosan, dextran, and alginate improve nanoparticle biocompatibility, biodegradability, and stability. These coatings also provide reactive functional groups for drug conjugation, targeted delivery, and biomedical applications.

### 5.5 Emerging combinational functionalization strategies for advanced multifunctional nanoparticles

Nanotechnology breakthroughs in recent years have led to a move from single molecule surface modification to a combination of functionalization methods, whereby various engineering methods are used to produce multifunctional nanoparticles that exhibit improved therapeutic action. Instead of using a single ligand or polymer, sophisticated nanoplatforms employ techniques like covalent coupling, grafting of polymers, ligand swapping, and biomimicking surface engineering to achieve all the above objectives (Kaymaz et al., 2023).

One possible approach is based on the development of bio-nanocomposites containing graphene oxide (GO), where its high specific surface area and oxygen functional groups are used for the immobilization of polymers, drugs, magnetic nanoparticles, and targeting agents simultaneously. In one particular approach, GO can be modified with polyethylene glycol (PEG) for enhanced biocompatibility, conjugated with target-specific ligands like folic acid or antibodies via chemical bonding, and combined with magnetic nanoparticles (Fe_3_O_4_) for performing magnetic hyperthermia and guided delivery. Concurrent drug loading by means of π-π interaction and electrostatic forces would allow the synergistic combination of chemotherapy with hyperthermia while reducing systemic toxicity (Azizi-Lalabadi and Jafari, 2021).

A new developing trend includes stimuli-responsive multifunctional nanoparticles in which the functional coating shows selective response to internal and external stimuli such as pH, enzymes, redox conditions, temperature, magnetic field, ultrasound, and near-infrared (NIR) illumination. The use of responsive polymers and inorganic nanoparticles provides a possibility to achieve precise spatiotemporal control of drug release and to implement diagnostics and photothermal or photodynamic therapy at the same time (Ahmad et al., 2026).

Moreover, biomimetic functionalization through cell membrane encapsulation, extracellular vesicles, peptides or protein corona can be incorporated with polymeric or inorganic nanoparticles to achieve immune escape, prolonged circulation time, and target-specific delivery. Thus, the future engineering of nanoparticles will include modular combination of various functionalization approaches in order to create a multi-purpose nanosystem for diagnosis, targeted therapy, drug delivery, and therapeutic monitoring (Feng et al., 2025).

### 5.6 Functionalization of carbon nanomaterials

#### 5.6.1 Carbon nanotubes

Carbon nanotubes (CNTs) possess excellent electrical, thermal, and mechanical properties; however, their practical applications are often limited by strong aggregation caused by π–π stacking interactions and van der Waals attractions between nanotube surfaces. To improve their stability and applicability, surface modification approaches are employed to introduce reactive and hydrophilic groups onto CNT surfaces.

One of the most used approaches is oxidative treatment using strong acids, which introduces oxygen-containing functional groups such as carboxyl, hydroxyl, and carbonyl groups onto CNT sidewalls and defect sites. Mechanistically, oxidation opens the nanotube ends and generates structural defect regions that serve as active sites for further surface interactions. The incorporation of these polar functional groups significantly enhances water dispersibility, sorption capacity, and compatibility with polymers and biomolecules. Surface-modified CNTs also exhibit improved interaction with metal ions, organic pollutants, drugs, and biological molecules, thereby expanding their applications in nanocomposites, environmental remediation, biosensing, catalysis, and drug delivery systems. Functionalization additionally improves interfacial adhesion between CNTs and polymer matrices, resulting in enhanced mechanical and electrical properties in composite materials (Asai et al., 2017).

#### 5.6.2 Graphene oxide functionalization

The graphene oxide (GO) comprises several oxygen-containing functional groups like epoxy, hydroxyl, and carboxyl groups that are situated on the basal planes and edges of the material; thus, it is an excellent material for functionalization purposes. These functional groups can enhance the adsorption of metal ions, conjugation with polymers, and catalytic efficiency while increasing the hydrophilic nature and dispersion of graphene oxide in aqueous solution. Besides, graphene oxide has excellent π-π interactions due to the availability of delocalized electrons in the graphitic basal plane. Owing to this reason, aromatic dyes, drugs, and organic pollutants can interact effectively with graphene oxide, resulting in efficient removal of contaminants. Graphene oxide has been widely investigated due to its large surface area and adsorption efficiency (Borane et al., 2024).

The different classes of nanoparticles exhibit unique physicochemical properties that influence their functionalization behaviour and biomedical potential. The different types of nanoparticles, their functionalization strategies, and major application advantages are summarized in Table 7.

**TABLE 7:** Different types of nanoparticles, their functionalisation strategies and its advantages.

Type of nanoparticles	Common examples	Functionalization strategies	Major advantages of functionalization	References
Metallic Nanoparticles	AuNPs, AgNPs, Platinum nanoparticles	Thiol chemistry; PEGylation; Silanization; Ligand exchange	• Improved colloidal stability • Enhanced biocompatibility • Targeted delivery • Reduced aggregation • Enhanced sensing and catalytic activity	Abdelkawi et al. (2023)
Semiconductor Nanoparticles	CdSe quantum dots, CdTe quantum dots, ZnO, TiO_2_ nanoparticles	Polymer coating; Core-shell modification; Ligand exchange	• Enhanced fluorescence stability • Improved photocatalytic efficiency • Better aqueous dispersibility • Reduced photodegradation	Wieszczycka et al. (2021)
Magnetic Nanoparticles	Fe_3_O_4_ nanoparticles, cobalt ferrite nanoparticles	Polymer/silica coating; PEGylation; Biomolecule conjugation	• Improved MRI contrast • Enhanced magnetic separation • Increased biocompatibility • Reduced aggregation	Kumar et al. (2023)
Carbon Nanotubes (CNTs)	Single-walled CNTs, multi-walled CNTs	Oxidative functionalization, polymer coating, biomolecule attachment	• Improved water dispersibility • Enhanced sorption capacity • Better polymer compatibility • Increased surface reactivity	Dubey et al. (2021)
Graphene Oxide Nanoparticles	GO, rGO	Oxygen-containing group modification, polymer conjugation, metal ion functionalization	• Enhanced adsorption capability • Improved dispersibility • Catalytic enhancement • Efficient pollutant removal	Gonçalves et al. (2024)
Silica Nanoparticles	Mesoporous silica nanoparticles, SiO_2_ nanoparticles	Silanization, amine functionalization, PEGylation	• Improved hydrophilicity • Surface reactivity • Drug loading capability • Enhanced stability	Gopi et al. (2016)
Polymeric Nanoparticles	PLGA nanoparticles, chitosan nanoparticles	PEGylation, hydrogel functionalization, polyelectrolyte coating	• Controlled drug release • Enhanced biocompatibility • Prolonged circulation time • Improved stability	Wang et al. (2013)
Lipid-Based Nanoparticles	Liposomes, solid lipid nanoparticles	PEGylation, ligand conjugation, surfactant coating	• Increased bioavailability • Reduced toxicity • Targeted drug delivery • Improved circulation stability	Aschmann et al. (2024)

CdSe QDs, cadmium selenide quantum dots; CdTe QDs, cadmium telluride quantum dots; ZnO NPs, zinc oxide nanoparticles; TiO_2_ NPs, titanium dioxide nanoparticles; Fe_3_O_4_ NPs, magnetite nanoparticles; CoFe_2_O_4_ NPs, cobalt ferrite nanoparticles; SWCNTs, single-walled carbon nanotubes; MWCNTs, multi-walled carbon nanotubes; GO, graphene oxide; rGO, reduced graphene oxide; MSNs, mesoporous silica nanoparticles; SiO_2_ NPs, silicon dioxide nanoparticles; PLGA NPs, poly (lactic-co-glycolic acid) nanoparticles; CS NPs, chitosan nanoparticles; SLNs, solid lipid nanoparticles.

Current research advances have contributed to extending the use of carbon-based nanoparticles from being just simple drug carriers to multifunctional theranostic platforms that can deliver drugs along with imaging and photothermal treatment and sensing applications. Graphene-based materials, carbon nanotubes, carbon dots, and graphene quantum dots possess superior drug loading, good functionalization, and stimuli-responsive drug release capabilities, which make them excellent candidates for precision nanomedicine (Alvandi et al., 2025; Wibowo and Anwar, 2026).

Although functionalization substantially enhances nanoparticle stability and targeting efficiency, balancing surface complexity with reproducible manufacturing and long-term biosafety remains a critical challenge.

## 6 Nanomaterials for diagnostics and bioimaging

### 6.1 Nano sensors for disease monitoring

**FIGURE 3:**
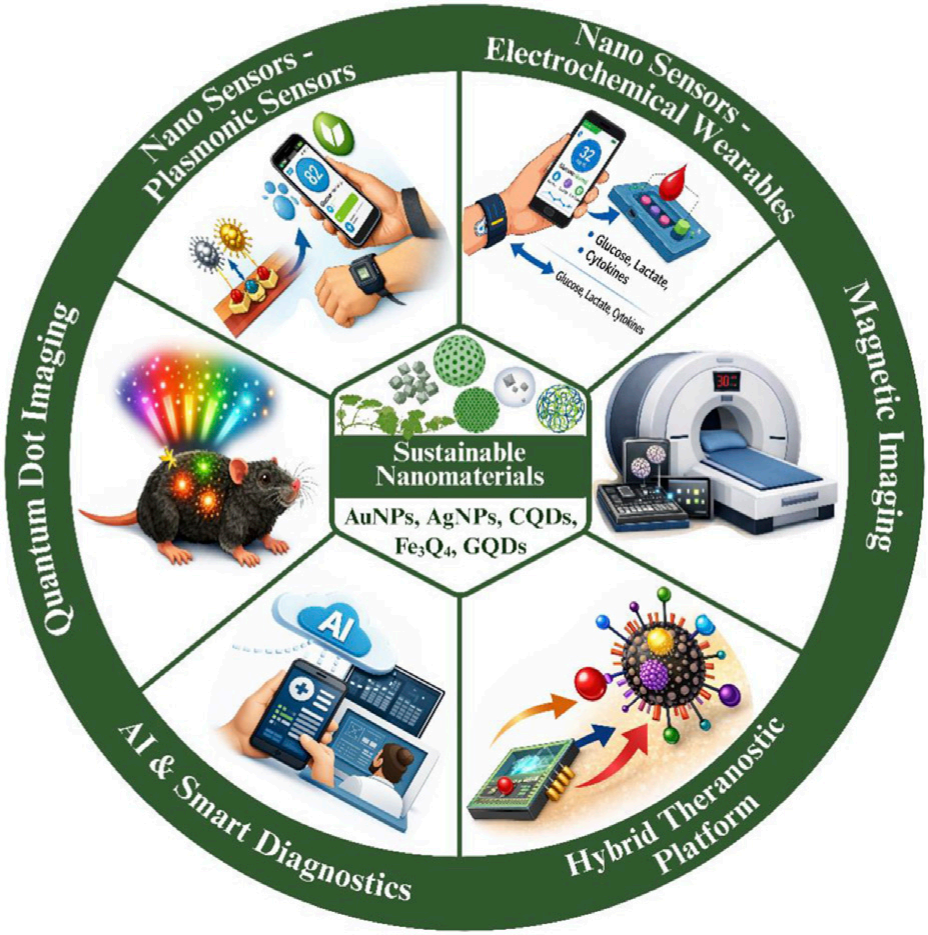
Green-synthesized nanomaterials for multimodal diagnostics and bioimaging.

Nanosensing platforms have gained attention as revolutionary technologies for detecting diseases in early stages, for real-time detection of biomarkers, and point-of-care diagnostics due to their increased sensitivity, miniaturization, and fast signalling capabilities (Figure 3). In sustainable nanomedicine, combination of metal nanoparticles obtained by green chemistry methods, nanographene-based materials, plasmonic nanoparticles, and electrochemical sensors has enabled the development of bioselective and biofriendly nano sensors for monitoring diseases. Green nanotechnological approaches are especially important in this area since biological nanomaterials possess lower toxicity, improved colloidal stability, easy functionalization, and environmental-friendly fabrication processes (Arghavani, 2025; Baran et al., 2025; Hassan et al., 2023; Kulkarni, 2022; Mandal et al., 2018; Noah, 2019; Shakitha and Devi, 2025). Among them, noble metal nanoparticles such as silver and gold nanoparticles mediated by microbes or plants are widely used as optical and electrochemical sensors. The unique size-dependent localized surface plasmon resonance (LSPR), electrical conductivity, and customizable surface chemistry of these nanoparticles allows their ultra-sensitive recognition of proteins, nucleic acids, glucose, inflammatory markers, and molecules from pathogens. Their excellent biocompatibility, along with the lack of harmful reducing reagents involved in the synthesis of the nanoparticles, makes it possible to utilize them directly in diagnostics (Baran et al., 2025; Mandal et al., 2018; Noah, 2019). Such plasmonic nano sensors are highly useful for the colorimetric or fluorescence-based detection of various diseases due to the specific optical changes induced by analytes (Baran et al., 2025; Noah, 2019). Graphene- and graphdiyne-based electrochemical analytical devices offer great promise for enhancing the performance of disease monitoring devices. The superb electron transport properties, mechanical flexibility, and high density of biomolecules in two-dimensional carbon nanomaterials make them suitable candidates for achieving signal amplification and low detection limits. Wearable graphene/graphdiyne-based electrochemical biosensors can now monitor glucose, lactate, inflammatory cytokines, and virus biomarkers in body fluids like sweat, saliva, and interstitial fluid (Kammarchedu et al., 2024). Furthermore, electrochemical nano biosensors made from green nanomaterials have gained increasing significance in rapid disease diagnosis owing to their portability, affordability, multiplexing capabilities, and ease of integration with microfluidic technologies. Green nano biomaterials like biogenic metallic nanoparticles, carbon dots, microbial nanocellulose, and nanocomposites of biopolymers boost the conductivity, catalysis, and specificity of electrodes (Grasso et al., 2019; Hassan et al., 2023; Thiruvengadam et al., 2025). Indeed, such platforms have been proved to be highly efficient in detecting various biomarkers associated with cancer, metabolites connected to diabetes, infection, and neurodegenerative markers. It is important to highlight that the development of novel sustainable smart biosensors combined with AI, Internet of Things (IoT), and cloud computing makes these platforms much more appealing. The AI signal processing enables accurate detection and prediction of diseases while IoT-based devices can provide an effective tool for deploying remote diagnostics and monitoring processes (Mallick et al., 2025; Thiruvengadam et al., 2025). Next-generation sensors represent promising technology that can address the demands of the underdeveloped regions which require rapid, sustainable, and efficient diagnostics. Significant progress has been made already. At the same time, there remain several challenges that need to be overcome concerning issues related to reproducibility, signal drifting, stability, biofouling, and lack of regulatory control (Arghavani, 2025; Hassan et al., 2023; Shakitha and Devi, 2025; Thiruvengadam et al., 2025). In order to unlock the full potential of nano biosensors in precision medicine, scientists have to find a way to achieve clinical validation of sustainable smart sensing systems.

### 6.2 Quantum dots and magnetic nanostructures

Quantum dots (QDs) and magnetic nanomaterials with high-resolution fluorescence microscopy, magnetism, and real-time molecular targeting are examples of such novel diagnostic techniques. Among these are semiconductor QDs, carbon quantum dots (CQDs), and graphene quantum dots (GQDs), all of which are highly efficient in fluorescence imaging owing to their size-related light emission capabilities and photo-luminescent stability (Figure 3) (Amiri et al., 2025; Gulati et al., 2025; Slimani and Hannachi, 2020). New developments have seen the development of hybridized nanoparticles for increased sensitivity to enable earlier detection of diseases. Moreover, fluorescent quantum nanomaterials enable *in vivo* tracking of therapeutic procedures and bio-markers, with GQDs being especially useful in cancer diagnosis and targeted cancer therapy owing to low toxicity levels (Amiri et al., 2025; Iannazzo et al., 2021).

Magnetic nanostructures, for example, Fe_3_O_4_ nanoparticles and super-paramagnetic iron oxide nanoparticles (SPIONs), is essential in improving contrast in MRI as well as magnetically assisted biosensing (Li et al., 2025; Priyadarshni et al., 2023; Slimani and Hannachi, 2020). The high magnetic susceptibility of such particles makes it possible to improve the sensitivity of T2-weighted MRI and GMR-based biosensors (Li et al., 2025; Mabarroh et al., 2022). Their use in medical imaging contributes to lesion contrast and tumor and inflammation localization (Gulati et al., 2025; Priyadarshni et al., 2023). An important development in the area includes the development of quantum dot-magnetic nanocomposites that integrate both fluorescence and magnetic imaging. Dual-modality systems such as mixed CdSe/ZnS QDs and Fe_3_O_4_ nanoparticles are used for DNA nanosensor applications due to their ability to capture targets effectively and achieve high fluorescence detection. Moreover, plant-synthesized Fe_3_O_4_ nanoparticles are considered one of the sustainable solutions for the field of nanomedicine as they are less toxic than regular reagents (Hushiarian et al., 2014; Mabarroh et al., 2022). However, challenges such as heavy metal toxicity in quantum dots, photobleaching, signal loss, and measurement standardization remain (Amiri et al., 2025; Li et al., 2025; Priyadarshni et al., 2023). Future developments may include carbon quantum dots, biomimetic ferrites, multifunctional theranostic agents, and AI-driven imaging reconstruction systems.

Nanocarrier-based delivery systems have similarly made substantial strides in the medical use of bioactive components extracted from herbs, which tend to be poorly soluble in water, have low bioavailability, and undergo quick metabolism. Nanocarrier formulations such as polymeric nanoparticles, lipid nanoparticles, nanofibers, nanoemulsions, dendrimers, and carbon-based nanoparticles can enhance the stability, controlled release, targeted delivery, and cellular uptake of phytochemicals like curcumin, resveratrol, quercetin, and epigallocatechin gallate. Modern scientific research shows that nanocarriers not only increase therapeutic effectiveness but also decrease toxicity, thus making it easier to apply them in clinics (Najafi et al., 2025).

The integration of sustainable nanomaterials with AI, wearable biosensors, and multimodal imaging represents a promising direction; however, clinical validation and regulatory standardization remain limited.

## 7 Regenerative nanomedicine and bioprinting

### 7.1 Nano-impregnated hydrogels for wound healing

The application of hydrogels with nanoparticles represents a novel approach in creating advanced wound dressings. These hydrogels combine their hygroscopic properties with the diverse functionalities of nanoparticles, resulting in benefits such as infection protection, angiogenesis stimulation, exudate adsorption, and sustained drug release. The unique 3D structure of hydrogels facilitates an aqueous environment, enhances gas exchange, and allows cell infiltration (Dam et al., 2023; Mallick et al., 2022; Wang et al., 2024). Nano-hydrogels are especially useful for angiogenesis and tissue regeneration by carrying various growth factors and bioactive components that aid in wound healing. For example, silver nanoparticle-impregnated marine collagen sponge hydrogels and Gelatin methacryloyl (GelMA) hydrogels promote improved mobility of fibroblasts and wound healing via better matrix interactions and sustained release of nanoparticles (Jahan et al., 2019; Sundar et al., 2023). The hybrid hydrogels loaded with metal nanoparticles possess remarkable antimicrobial effects, especially useful for wound infections and burn cases where bacterial colonization in biofilms occur regularly. AgNPs, CuO NPs, and ZnO NPs possess antibacterial activities based on different mechanisms such as production of Reactive oxygen species (ROS) and disruption of the cell membranes (Hebeish and Sharaf, 2015; Mallick et al., 2022; Pangli et al., 2021). The nanocomposites are capable of eliminating Gram-positive and Gram-negative bacteria, thus preventing further infection. Furthermore, the release of drugs using hydrogel systems improves the efficacy of the treatments, with the processes of releasing being regulated through pH, enzyme activity, moisture, or diffusion (Mallick et al., 2022; Wang et al., 2024). Metal nanoparticles can be used to increase the antibacterial activity along with the robustness and sensitivity of hydrogels to different stimuli (Jahan et al., 2019; Pangli et al., 2021). Nanogreen chemistry has made advances in the development of environmentally friendly and biocompatible dressings, especially when using silver nanoparticles and marine collagen sponges to achieve controlled delivery of drugs and tissue regeneration (Sundar et al., 2023). Despite remarkable advancements in the field, there are several issues that remain unaddressed regarding cytotoxicity, large-scale production, and regulatory aspects for hybrid materials (Dam et al., 2023; Pangli et al., 2021; Wang et al., 2024). Another interesting opportunity for smart hydrogels is the combination with wound diagnostic systems that involve biosensors and AI.

### 7.2 Carbon- and polymer-based scaffolds

Carbon-based and polymeric scaffolds have gained much value in regenerative nanomedicine owing to their structural similarity with characteristics of native Extracellular matrix (ECM). The scaffold provides a 3-dimensional structure for cell attachment, transport of nutrients, and regeneration (Eivazzadeh-Keihan et al., 2024; Ku et al., 2013). Carbon nanostructures like graphene oxide (GO), reduced graphene oxide (rGO), CNTs, and nanodiamonds are incorporated into polymer systems to improve the properties of scaffolds owing to their high mechanical strength, high surface area, and conductivity (Eivazzadeh-Keihan et al., 2024; Ku et al., 2013). They allow better interactions of cells and formation of ECM nanostructures that is particularly valuable in bone, neuronal, and skin tissue engineering because substrate topography affects the behavior of cells (Holt et al., 2017; Ku et al., 2013).

Natural and artificial biocompatible and biodegradable scaffolds based on polymeric materials (collagen, chitosan) and synthetic polymers (polycaprolactone) are widely used due to their properties. Nevertheless, the scaffolds made of pure material do not always show sufficient mechanical stability and bioactivity which can be solved by using carbon nanomaterials (Eivazzadeh-Keihan et al., 2024; Gobi et al., 2021). For instance, electrospun Polyvinyl alcohol (PVA)/reduced graphene oxide scaffolds showed improved hemocompatibility and fibroblast proliferation, while carbon nanotube-infused scaffolds facilitate wettability and controlled drug delivery. In bone tissue engineering, composite materials improve osteoblast behaviour and create a conducive environment for mineralization. For soft tissue repair, carbon composites enhance angiogenesis and accelerate wound healing, showcasing the versatility of carbon nano constructs in optimizing scaffold performance based on specific tissue needs (Holt et al., 2017; Lekshmi et al., 2020; Sadat et al., 2022; Vedhanayagam et al., 2019). However, there are still some issues concerning dose-related nanocarbon toxicity, inflammation, biodegradation over time, and dispersion of nanofillers within the polymer matrix (Eivazzadeh-Keihan et al., 2024; Ku et al., 2013; Stocco et al., 2023). Future design of such scaffolds should consider the development of environmentally friendly fabrication techniques, biomimetic surface modifications, intelligent conductive scaffolds, and three- or four-dimensional printing of polymer-carbon composites.

Nanofibers produced by electrospinning technology represent a very exciting delivery system at the nanoscale due to their large surface area, porosity and capacity to imitate the natural extracellular matrix. These properties facilitate the encapsulation and release of various bioactive components like antibiotics, growth factors, phytochemicals, peptides, and molecules obtained from stem cells. Hybrid nanofibers produced through biodegradable polymers such as poly (ε-caprolactone), polyvinyl alcohol and chitosan enhance the physical properties, moisture retention, antimicrobial potential and cell adherence, making them an ideal choice for wound healing and tissue engineering applications. It was recently found out that squalene-based hybrid nanofiber matrices promote faster wound healing via enhancement of angiogenesis, collagen formation and proliferation of fibroblasts along with decreasing inflammation in wounds (Al-Abduljabbar and Farooq, 2022; Cho et al., 2025; Noori et al., 2025).

### 7.3 Nano-based bioinks for 3D and 4D bioprinting

Nanomaterial-containing bioinks represent an advanced category of biomaterials utilized in three- and four-dimensional bioprinting, addressing limitations of traditional hydrogel bioinks, such as insufficient mechanical stability and poor biological activity. By integrating nanoparticles, nanofibers, nanoclays, graphene materials, nanocellulose, and biomimetic nanocrystals, these bioinks enhance critical properties including viscosity, self-recovery, and shape retention, which are vital for effective extrusion processes (Chakraborty et al., 2021; Chimene et al., 2020; Municoy et al., 2025). Nanomaterials reinforce the bioink structure, bolstering mechanical resilience through interactions with polymer chains, including entanglements and cross-linking reactions (Chakraborty et al., 2021; Chimene et al., 2020). For example, bioinks with montmorillonite nanoclay have a higher strength in both tensile and compression, as well as better printability, making it possible to fabricate strong scaffolds (Wu, 2020). Similarly, nanocellulose improves rheological behaviour due to the high aspect ratio and viscoelastic properties (Rana and Thakur, 2023). Moreover, such bioinks make bioactive environment that helps cell adhesion and differentiation, with certain nanoparticles being able to simulate natural bone minerals and thus be useful in bioprinting of bone and osteochondral tissues because of osteogenic activity (Cai et al., 2023; Fischetti et al., 2023). Finally, graphene oxide bioinks are used for applications in the field of neurobiology, cardiology, and myogenesis due to cellular affinity and possibility of forming conductive interface (Li et al., 2022). Bioinks based on decellularized extracellular matrix, nanoclay, and sodium alginate prove to be hemocompatible and have desirable biomechanical properties in case of vascular scaffolds (Zhu et al., 2025).

A major Frontier that exists within 4D bioprinting involves the field of tissue engineering where nano-based bioinks, which are able to morph based on external stimulation such as temperature, pH, ionic concentration, magnetic field, electric impulses, and near-infrared radiations have been developed (Chakraborty et al., 2021). The advantage of these bioinks compared to traditional 3D printed scaffolds is that they are programmable and allow post-print morphogenesis which enhances the process of tissue regeneration in dynamic conditions. Some of the major ingredients that are used include magnetosomes and iron oxide nanoparticles for magnetic stimuli while graphene oxide and plasmonic nanoparticles are used for photoelectric and photothermal stimuli. These bioinks can be used in designing scaffolds that are suitable for vascular grafts, cartilage implants, responsive wound healing, and musculoskeletal tissues among others. There are challenges facing this technology including toxicity, clogging, heterogeneity, problems in vascularizing tissues, and inability to print different types of bioinks (Chakraborty et al., 2021; Chimene et al., 2020; Municoy et al., 2025).

Future advances are expected to focus on programmable biomaterials, electrospun nanofibers, multifunctional bioinks, and patient-specific scaffolds capable of dynamic tissue regeneration under clinically relevant conditions.

## 8 Challenges and future perspectives

Although green nanomedicine has shown promising outcomes in areas such as drug delivery, antimicrobial therapy, diagnostics, and tissue engineering, several limitations still need to be addressed before these systems can be translated into routine clinical practice. The major concerns is the inconsistency associated with biological synthesis routes. Since plant extracts, microbial cultures, and biomolecules vary depending on source and environmental conditions, maintaining uniform nanoparticle characteristics during large-scale production remains difficult (Gupta et al., 2023; Iravani, 2011). In many cases, even small variations in synthesis conditions can influence particle size, stability, and biological performance.

Another important issue is the limited understanding of the exact molecular mechanisms involved during nanoparticle bio-reduction and stabilization. Although many studies report successful green synthesis, the interaction between biomolecules and nanoparticle surfaces is still not fully understood. Although phytochemicals such as polyphenols, flavonoids, and proteins are known to facilitate nanoparticle formation, the precise role of individual functional groups remains unclear. For example, how hydroxyl, carboxyl, and amino groups influence metal ion reduction, particle nucleation, and the final size or shape of nanoparticles has not been fully established. A better understanding of these interactions could improve process reproducibility and support the design of nanomaterials with more predictable physicochemical and biological properties (Ayub et al., 2025; Iravani, 2011). In addition, concerns related to long-term toxicity, biodistribution, regulatory approval, and industrial scalability continue to slow down the clinical translation of several promising nanoplatforms (Bobo et al., 2016; Mitragotri et al., 2015). Commercialization presents another major challenge for green nanomedicines. The absence of standardized manufacturing protocols and internationally accepted regulatory guidelines continues to hinder their wider adoption. Variability in raw biological materials and synthesis conditions makes quality control difficult, while large-scale production requires consistent product quality, cost-effectiveness, and compliance with regulatory standards. Addressing these issues will be essential for successful clinical translation and commercial adoption (Bobo et al., 2016; Gupta et al., 2023).

Despite these challenges, the future scope of sustainable nanomedicine remains highly encouraging. The integration of AI, smart biosensors, theranostic systems, and nano-enabled bioprinting is expected to improve the design and functional performance of next-generation nanomaterials (Chakraborty et al., 2021; Mallick et al., 2025). Similarly, the development of stimuli-responsive biomaterials and multifunctional nano-bioinks may open new possibilities in precision medicine and regenerative healthcare. Although considerable progress has been made, several questions still remain unanswered. There is still substantial scope for improving our understanding of green nanoparticle synthesis. More work is needed to explain how different biomolecules influence nanoparticle formation and why similar biological systems often produce particles with different properties. Standardized synthesis procedures and detailed biosafety studies will also be necessary to improve reproducibility and support clinical translation. At the same time, emerging computational approaches, including AI, may help optimize synthesis conditions and accelerate the development of application-specific nanomaterials (Mallick et al., 2025). Bringing green nanomedicine into routine clinical practice will require coordinated efforts from researchers, clinicians, industry, and regulatory agencies. Such interdisciplinary research and collaboration will be essential to overcome the remaining challenges associated with standardization, large-scale production, and regulatory approval.

## 9 Conclusion

The convergence of green chemistry and nanomedicine has created a transformative framework for developing sustainable, biocompatible nanotherapeutic systems. Green synthesis methods using plants, microorganisms, enzymes, and biopolymers present environmentally friendly alternatives to traditional nanoparticles, enhancing biological performance and colloidal stability, while reducing systemic toxicity. Functionalized nanomaterials such as metallic nanoparticles, metal oxides, magnetic nanomaterials, carbon-based materials, polymer nanoparticles, nanozymes, and nanocomposites have shown potential in various biomedical applications, including drug delivery, antimicrobial therapy, cancer treatment, and tissue engineering. Techniques like covalent modification, ligand exchange, PEGylation, and bioconjugation improve therapeutic efficiency through better targeting, stability, and controlled release. The emergence of regenerative nanotechnologies and their integration with intelligent biosensors and theranostic signals a breakthrough in dynamic tissue engineering and disease management. Despite experimental successes, challenges such as understanding reduction mechanisms, achieving batch reproducibility, addressing long-term nanotoxicity, and regulatory uncertainties must be resolved for clinical and industrial adoption. Future efforts should focus on biological processes, production scaling, surface engineering, and comprehensive safety studies. Collaboration across nanotechnology, biotechnology, materials science, and medicine is essential to accelerate the translation of green nanomedicine into practice, promising a sustainable and effective future for healthcare.
